# Segregation Analysis of Genotyped and Family-Phased, Long Range MHC Classical Class I and Class II Haplotypes in 5 Families With Type 1 Diabetes Proband in the United Arab Emirates

**DOI:** 10.3389/fgene.2021.670844

**Published:** 2021-06-30

**Authors:** Guan K. Tay, Halima Al Naqbi, Aurélie Mawart, Zahrah Baalfaqih, Anoud Almaazmi, Asma Deeb, Habiba Alsafar

**Affiliations:** ^1^Center for Biotechnology, Khalifa University of Science and Technology, Abu Dhabi, United Arab Emirates; ^2^Department of Biomedical Engineering, Khalifa University of Science and Technology, Abu Dhabi, United Arab Emirates; ^3^College of Medicine and Health Sciences, Khalifa University of Science and Technology, Abu Dhabi, United Arab Emirates; ^4^Faculty of Health and Medical Sciences, University of Western Australia, Nedlands, WA, Australia; ^5^School of Medical and Health Sciences, Edith Cowan University, Joondalup, WA, Australia; ^6^Department of Endocrinology, Mafraq Hospital, Abu Dhabi, United Arab Emirates

**Keywords:** type 1 diabetes, major histocompatibility complex, UAE, Arab, DR3/DR4 haplotypes

## Abstract

The classical Human Leucocyte Antigen (HLA) class II haplotypes of the Major Histocompatibility Complex (MHC) that are associated with type 1 diabetes (T1D) were identified in five families from the United Arab Emirates (UAE). Segregation analyses were performed on these 5 families with the disease, 3 with one child and 2 with 2 children diagnosed with T1D. Three HLA-DR4 haplotypes were identified: HLA- DRB1^∗^04:01:01-DQB1^∗^03:02:01:01; HLA- DRB1^∗^04:02:01- DQB1^∗^03:02:01; and HLA -DRB1^∗^04:05:01-DQB1^∗^02:02:01:02. All have previously been identified to be associated with T1D in studies of the Arabian population. In the 10 parents from the 5 families, 9 had at least one HLA-DR4 and HLA-DR3 haplotype which potentially increases the risk of T1D. Of these 9 parents, 3 were heterozygous for HLA-DR4/HLA-DR3 and one was homozygous for HLA-DR3. Two haplotypes that were identified here extend to the HLA class I region were previously designated AH8.2 (HLA -A^∗^26-B^∗^08-DRB1^∗^03) and AH50.2 (HLA -C^∗^06-B^∗^50-DRB1^∗^03:01-DQ^∗^02) and associated with diabetes in neighboring North Indian populations. This study provides examples of MHC haplotype analysis in pedigrees to improve our understanding of the genetics of T1D in the understudied population of the UAE.

## Introduction

Type 1 diabetes (T1D), also known as Insulin Dependent Diabetes Mellitus (IDDM), is a complex autoimmune disorder that results from the autoimmune destruction of insulin-producing beta cells in the pancreas. Susceptibility to T1D is due to interactions between genetic and environmental factors (Noble, 2015). Universally, the strongest genetic risk has been attributable to genes of the Major Histocompatibility Complex (MHC), and the strongest association has been attributed to the classical Human Leukocyte Antigens (HLA), in particular the HLA class II genes (Noble et al., 1996, 2000; Cucca et al., 2001; Cruz et al., 2004; Erlich et al., 2008; Varney et al., 2010). The HLA genes are categorized into class I and class II genes. HLA class I (HLA-A, HLA-C, HLA-B) present endogenous or cytosol derived peptides, whereas HLA class II (HLA-DR, HLA-DQ, HLA-DP) present extracellular antigens. These genes are polymorphic with a numerical system denoting alleles (e.g., HLA-B^∗^08 or HLA-DRB1^∗^03). Due tight linkage disequilibrium MHC across the region (Kulski et al., 2021), the linked class I and II genes exist as conserved extended (Alper et al., 1989) or ancestral (Dawkins et al., 1999) haplotypes. For example, the ancestral haplotype (AH) denoted as AH8.1 carries the allele combination: HLA -A^∗^01-B^∗^08-DRB1^∗^03.

The association between HLA class II haplotypes and the increased risk of T1D is well known in patients of European or Caucasian descent (Pociot and McDermott, 2002; Larsen and Alper, 2004; Awdeh et al., 2006; Noble, 2015). The highest risk has been identified in a specific set of heterozygote genotypes consisting of both HLA-DR3 and HLA-DR4, including DR3, DQ2/DR4, DQ8 genotypes.

Other haplotypes have shown a protective effect against the disease (Pociot and McDermott, 2002; Larsen and Alper, 2004; Noble, 2015). A common T1D protective haplotype is HLA -DRB1^∗^15:01-DQA1^∗^01:02-DQB1^∗^06:02 (also known as DR2), commonly found in Caucasians (Noble, 2015). Other haplotypes that have also been shown to be protective against the disease include HLA -DRB1^∗^11:04-DQA1^∗^0501-DQB1^∗^03:01, HLA -DRB1^∗^07:01-DQA1^∗^02:01-DQB1^∗^03:03, and HLA -DRB1^∗^14:01-DQA1^∗^01:01-DQB1^∗^05:03 (Noble, 2015).

Patients of Arabian ancestry share several MHC haplotypes which confer either susceptibility or protection to T1D with other ethnic groups (Al Naqbi et al., 2021). However, there are also other disease-associated MHC haplotypes in Arabs that are distinct from other ethnic groups. Research involving genetic predisposition to T1D in ethnic groups of Arabia has been sparse and efforts have been few and far between. Consequently, the role of genetic and non-genetic components remains unclear. Nevertheless, HLA-DRB1^∗^03, HLA-DRB1^∗^04 and HLA-DQB1^∗^02 that are positively associated with increased risk of T1D in African Arabs (Hajjej et al., 2019) is consistent with a meta-analysis study by Hamzeh et al. (2016) that showed significant increases in T1D risk in patients with the HLA-DRB1^∗^03:01, HLA-DRB1^∗^04:01, HLA-DRB1^∗^04:02, and HLA-DRB1^∗^04:05 alleles in a separate Arabian study (Hamzeh et al., 2016). The MHC haplotype, HLA -DRB1^∗^04-DQB1^∗^02, has also been shown to be associated with T1D in Saudi Arabians (Al-Hussein et al., 2003). HLA-DR3 and HLA-DR4 appears the most common subtypes in T1D disease association studies involving Arabian populations (Al-Hussein et al., 2003; Al-Harbi et al., 2004; Al-Jenaidi et al., 2005; Al-Herbish et al., 2008). However, there are a number of ethnic specific differences (Al Naqbi et al., 2021). Of note, a study by Hajjej et al. (2018) reported that HLA-DRB1^∗^03/HLA-DRB1^∗^04 genotype showed the highest risk of T1D development (Hajjej et al., 2018). This is contrary to the findings in Asians described in a review by Park and Eisenbarth (2001), which suggested that high risk was associated with HLA-DR4 subtypes in HLA-DR4/X, whereas protective HLA-DR4 subtypes were observed mainly in the HLA-DR4/HLA-DR3 genotypes. Nevertheless, in a separate study in Tunisia, a significant increase in homozygosity of the HLA -DRB1^∗^03:01:01-DQB1^∗^02:01 haplotype was observed in T1D subjects (Stayoussef et al., 2009a).

Despite the strong contribution of HLA class II haplotypes to T1D, there is interest in additional genes in the MHC due to the tight linkage disequilibrium that exists across the region (Kulski et al., 2021). For example, the HLA -DR3-DQ2 haplotype can be extended toward the class I region to include HLA -A1-C7-B8 (Degli-Esposti et al., 1992a, b) as well as other loci. In North India, diabetes has been associated with the extended MHC haplotypes from class I (HLA -A26-C7-B8), through to the central region carrying inflammatory and complement genes to the class II region (HLA -DR3-DQ2) (Mehra et al., 2002, 2007; Kanga et al., 2004). In fact, using microsatellite markers from the telomeric end of the MHC, haplotypes may be conserved in their ancestral form to at least as far as D6S105, which is around 2 megabases telomeric of HLA-A loci (Tay et al., 1997), making MHC ancestral haplotypes (AHs) a conserved region of genomic sequence spanning around 6–8 megabases from the HLA class II region and beyond the HLA class I region up to and including the HFE gene (Cattley et al., 2000).

Despite anecdotal data suggesting T1D is on the rise in the UAE, there is a dearth of information about the prevalence of the disease in the population. A recent review revealed that most of the research in the country focused mainly on type 2 diabetes (T2D) with only 14 of 314 published articles (accessed in 2020) presenting data on T1D (Shieb et al., 2020).

In this study, we performed HLA typing and constructed 8 locus MHC haplotypes using segregation analyses in 5 families to define the haplotypes that are common in the UAE, with the goal of identifying any haplotypes that were shared among individuals with T1D in different families.

## Materials and Methods

### Recruitment

The parents of patients that were diagnosed with T1D were approached during a routine follow up visit to the Department of Endocrinology at Mafraq Hospital in Abu Dhabi, UAE. The family and the patients were initially briefed about the project and invited to be part of the study. The study aimed to characterize HLA alleles and MHC haplotypes in families with a T1D proband. All participants who volunteered to be part of the study signed a consent form that was approved by the Institutional Review Board (IRB) committee of Mafraq Hospital (MAF-REC_07/2016_04). Assent was obtained from the parents of participants who were too young to provide consent.

### Sample Collection

Each sample was collected using an individual Oragene-DNA kit (Genotek, Ottawa, Canada) according to the guidelines provided by the manufacturer.

### Diagnosis of T1D

The diagnostic criteria for type 1 diabetes were based on the International Society for Paediatric Endocrinology and Diabetes (ISPAD) (Mayer-Davis et al., 2018). Patients were initially diagnosed with diabetes after presentation with hyperglycemia with or without diabetes ketoacidosis. Specifically, all children presented with classic symptoms of diabetes with plasma glucose concentration of more than 200 mg/dL and had HbA1c levels of more than 6.5%. All returned auto-antibody positive results and had low C-peptide, in keeping with T1D. All children had a minimum of one positive autoantibodies of either anti GAD, anti-IA-2 or IAA antibodies. Table 1 presents clinical data on the T1D subjects of this study.

**TABLE 1:** Clinical data on the T1D subjects of this study.

Patient		Birth year	Gender	Age at diagnosis (years)	Duration of diabetes (years)	Diabetes presentation	Affected siblings	HbA1c (%)
F2	I.i	2000	M	4	15	DKA	No	9.4
F14	II.iii	2003	F	8	8	DKA	Yes	8.0
	II.iv	2001	M	13	5	Hyperglycemia	Yes	11.0
F22	II.iii	2010	M	1	8	Hyperglycemia	No	10.3
F23	II.iii	2010	M	2	7	DKA	No	8.2
F24	II.iii	2009	M	7	3	Hyperglycemia	Yes	8.9
	II.iv	2000	F	7	12	Hyperglycemia	Yes	9.6

*DKA: Diabetic Ketoacidosis, HbA1c: Haemoglobin A1C.*

### DNA Extraction and HLA Typing

Each patient’s DNA was extract using the reagents provided with the Oragene-DNA kit (Genotek, Ottawa, Canada), following the manufacturer guidelines. The quality of DNA was assessed by visualizing an aliquot on a 0.8% agarose gel. The concentration and quantity of each DNA sample was determined by a fluorometric method using the dsDNA broad range 2 -point assay (DeNovix, Wilmington, United States). Each DNA sample was diluted to 10 ng/μl and used for the preparation of the sequencing library. The TruSight HLA library kit covering 11 loci (Illumina, San Diego, United States) was used in accordance with the manufacturer’s recommendations. The size distribution of each sample library was check before pooling using a fragment analyzer (Agilent, Santa Clara, United States) with a high sensitivity NGS fragment analysis kit. The final library of 24 samples were quantified with qPCR method using KAPA library quantification kit ROX low (Kappa Biosystems, Wilmington, United States) on a ViiA 7 (Applied Biosystems, Foster City, United States). After dilution, libraries were sequence with MiSeq sequencer (Illumina, San Diego, United States) with a reagent micro kit. The HLA alleles for each sample was assigned after sequence analysis with the Assign TruSight HLA Analysis software v2.1.0.943 using HLA references release version 3.29 (Illumina, San Diego, United States).

### Segregation Analysis

Segregation analysis by family study was independently performed by 3 of the co-authors and all haplotypes assigned were concordant between these 3 individuals. The same haplotypes in each family were identical by descent.

## Results

**FIGURE 1:**
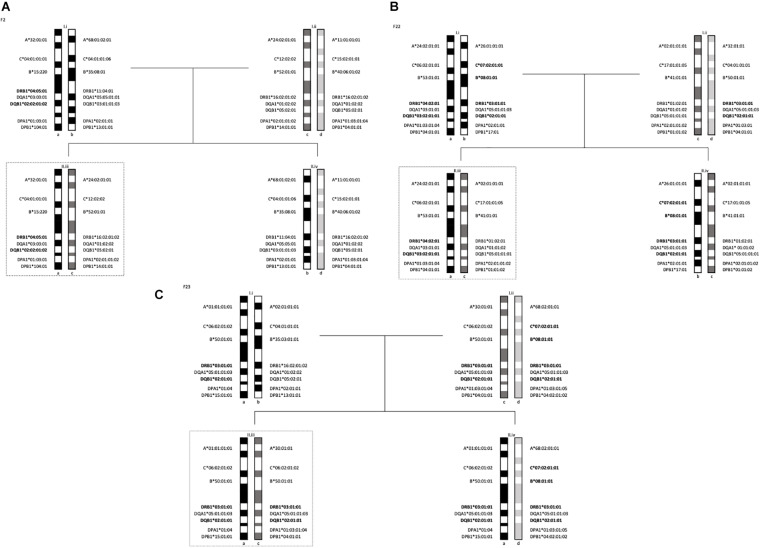
Segregation analysis of the 5 families with one T1D subject showing the MHC long-range haplotypes. In dotted lines are offsprings diagnosed with T1D. **(A)** Family 2 haplotypes, II.iii is diagnosed with T1D. **(B)** Family 22 haplotypes, II.iii is diagnosed with T1D. **(C)** Family 23 haplotypes, II.iii is diagnosed with T1D.

Segregation analyses were performed on 5 families. In each family, there was either 1 or 2 members diagnosed with T1D. In family F2 (Figure 1A), one offspring diagnosed with T1D at the age of 4 years (F2-II.iii) inherited one haplotype from the father (F2-I.i) marked by HLA -DRB1^∗^04:05:01-DQB1^∗^02:02:01:02. The HLA-DQA1^∗^03:03:01 allele of this haplotype is consistent with the T1D susceptible combination of HLA -DRB1^∗^04:05-DQA1^∗^03-DQB1^∗^02 described in Egyptian patients (El-Amir et al., 2015). The second haplotype, inherited by descent from the mother, was HLA- DRB1^∗^16:02:01:02-DQB1^∗^ 05:02:01.

**FIGURE 2:**
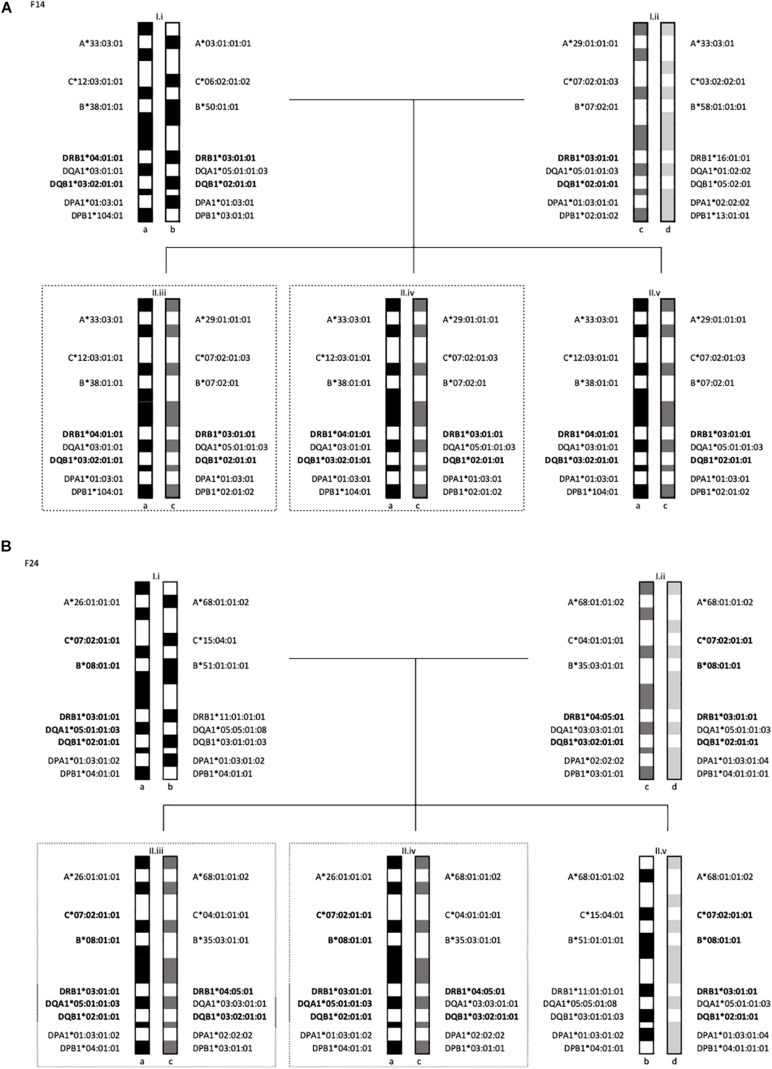
Segregation analysis of the 5 families with multiple T1D subjects showing the MHC long-range haplotypes. In dotted lines are offsprings diagnosed with T1D. **(A)** Family 14 haplotypes II.iii and II.iv are diagnosed with T1D. **(B)** Family 24 haplotypes, II.iii and II.iv are diagnosed with T1D.

In family F24 (Figure 2B), two (F24-II.iii and F24-II.iv) of the 3 offspring were heterozygous for HLA-DR4 and HLA-DR3 haplotypes. Specifically, the inherited haplotypes in this family were HLA- DRB1^∗^04:05:01-DQB1^∗^03:02:01:01 and HLA -DRB1^∗^03:01:01-DQB1^∗^02:01:01.

All three offspring in family F14 (Figure 2A) were HLA-identical for the entire MHC, marked by two HLA class II haplotypes: HLA -DRB1^∗^04:01:01-DQB1^∗^03:02:01:01-DPB1^∗^104:01 and HLA -DRB1^∗^03:01:01-DQB1^∗^02:01:01-DPB1^∗^02:01:02. Two of the 3 offspring (F14-II.iii and F14-II.iv) were diagnosed with diabetes, one at age 8 and the second at age 13. The third offspring (F14-II.v) remained unaffected at last follow-up at age 14 years old. Of note is the fathers’ genotype (ab), which is different to that of his children (ac) but is marked by exactly the same allelic combinations at HLA-DRB1 and HLA-DQB1. The “b” and “c” haplotypes differ at HLA-DPB1, HLA-B, HLA-C, and HLA-A (see Figure 2A).

The two children in family F22 (Figure 1B) shared one haplotype HLA -DR1-DQ5, but the second haplotype was different. One child carried HLA -DRB1^∗^04:02:01-DQB1^∗^03:02:01:01 (II.iii) and the other carried HLA -DRB1^∗^03:01:01:01-DQB1^∗^02:01:01 (II.iv). The HLA-DRB1^∗^04:02 allele is linked to HLA-DQB1^∗^03:02 and has been shown to be significantly associated to T1D in a multi-ethnic meta-analysis by Thomson et al. (2007). This HLA-DR4 subtype has also been found to be transmitted in German and Belgian families with T1D (Donner et al., 2000) and associated with T1D in Hispanics (Rewers et al., 2003). The offspring with the HLA-DR4 haplotype (F22-II.iii) was diagnosed with T1D at the age of 1 year.

Finally, the child (F23-II.iii) that was diagnosed with T1D at the age of 2 years carried the ancestral haplotype (AH) AH50.2 and was homozygous for the HLA class II haplotype: HLA -DRB1^∗^03:01:01:01-DQB1^∗^02:01:01. This is a susceptible haplotypes described in studies of T1D in the populations of Lebanon (Al-Jenaidi et al., 2005), Bahrain (Al-Jenaidi et al., 2005), Morocco (Drissi Bourhanbour et al., 2015) and Tunisia (Stayoussef et al., 2009a). The mother in this family (F23-I.ii) was also homozygous for the same two HLA-DR3 and -DQ2 subtypes, however, she did not share the same HLA-DP subtype (see Figure 1C).

In three (F22, F23, F24) of the 5 families, the HLA -DR3-DQ2 haplotypes extend to the HLA class I region, carrying HLA-B^∗^08 and HLA-C^∗^07 (Table 2). The ancestral haplotype designated AH8.1, marked by HLA-A1, HLA-C7, HLA-B8, HLA-DR3, and HLA-DQ2 has previously been shown to be associated with T1D (Degli-Esposti et al., 1992a), primarily in Caucasians. The HLA -B8-DR3 haplotypes in Family F22 and F24 is linked to HLA-A^∗^26 (Table 2) and has been recognized as a conserved haplotype referred to as AH8.2, which is common in the Asian Indian (Witt et al., 2002). There were two others linked to HLA-A^∗^68 which to the best of our knowledge is not commonly associated with HLA -B8-DR3. Subtypes of this allele is higher in certain native American, South Asian, Central Asian and African groups and it is not surprising that it is found in the Middle Eastern population of the UAE as it has been postulated that these have been sourced from Africa via migration through specific Asian routes to the New World.

**TABLE 2:** Segregation analysis of HLA haplotypes from 5 families reveals the presence of extended haplotypes in different and apparently unrelated families.

Family	Haplotype	HLA-A	HLA-C	HLA-B	HLA-DRB1	HLA-DQB1	HLA-DPB1	AH
F14	c	A*29:01:01:01	C*07:02:01:03	B*07:02:01	DRB1*03:01:01	DQB1*02:01:01	DPB1*02:01:02	
F22	b	A*26:01:01:01	C*07:02:01:01	B*08:01:01	DRB1*03:01:01	DQB1*02:01:01	DPB1*17:01	8.2
F24	a	A*26:01:01:01	C*07:02:01:01	B*08:01:01	DRB1*03:01:01	DQB1*02:01:01	DPB1*04:01:01	8.2
F24	d	A*68:01:01:02	C*07:02:01:01	B*08:01:01	DRB1*03:01:01	DQB1*02:01:01	DPB1*04:01:01:01	8.2R
F23	d	A*68:02:01:01	C*07:02:01:01	B*08:01:01	DRB1*03:01:01	DQB1*02:01:01	DPB1*04:02:01:02	8.2R
F2	a	A*32:01:01	C*04:01:01:01	B*15:220	DRB1*04:05:01	DQB1*02:02:01:02	DPB1*104:01	
F23	b	A*02:01:01:01	C*04:01:01:01	B*35:03:01:01	DRB1*16:02:01:02	DQB1*05:02:01	DPB1*13:01:01	
F24	c	A*68:01:01:02	C*04:01:01:01	B*35:03:01:01	DRB1*04:05:01	DQB1*03:02:01:01	DPB1*03:01:01	
F2	b	A*68:01:02:01	C*04:01:01:06	B*35:08:01	DRB1*11:04:01	DQB1*03:01:01:03	DPB1*13:01:01	
F14	a	A*33:03:01	C*12:03:01:01	B*38:01:01	DRB1*04:01:01	DQB1*03:02:01:01	DPB1*104:01	
F2	d	A*11:01:01:01	C*15:02:01:01	B*40:06:01:02	DRB1*16:02:01:02	DQB1*05:02:01	DPB1*04:01:01	
F22	c	A*02:01:01:01	C*17:01:01:05	B*41:01:01	DRB1*01:02:01	DQB1*05:01:01:01	DPB1*01:01:02	
F22	d	A*32:01:01	C*04:01:01:01	B*50:01:01	DRB1*03:01:01	DQB1*02:01:01	DPB1*04:01:01	50.2R
F23	a	A*01:01:01:01	C*06:02:01:02	B*50:01:01	DRB1*03:01:01	DQB1*02:01:01	DPB1*15:01:01	50.2
F14	b	A*03:01:01:01	C*06:02:01:02	B*50:01:01	DRB1*03:01:01	DQB1*02:01:01	DPB1*03:01:01	50.2
F23	c	A*30:01:01	C*06:02:01:02	B*50:01:01	DRB1*03:01:01	DQB1*02:01:01	DPB1*04:01:01	50.2
F24	b	A*68:01:01:02	C*15:04:01	B*51:01:01:01	DRB1*11:01:01:01	DQB1*03:01:01:03	DPB1*04:01:01	
F2	c	A*24:02:01:01	C*12:02:02	B*52:01:01	DRB1*16:02:01:02	DQB1*05:02:01	DPB1*14:01:01	
F22	a	A*24:02:01:01	C*06:02:01:01	B*53:01:01	DRB1*04:02:01	DQB1*03:02:01:01	DPB1*04:01:01	
F14	d	A*33:03:01	C*03:02:02:01	B*58:01:01:01	DRB1*16:01:01	DQB1*05:02:01	DPB1*13:01:01	

*“R” in AH column denotes recombinant.*

In bold are the group of alleles that makeup the designated ancestral haplotype in column AH.

* sign indicates that typing is performed by a molecular method.

In four of the five families studied, at least one parent carried two haplotypes that at face value increase the risk of T1D (The one only exception being F2). The mother of family F23 (F23-I.ii) was homozygous for HLA -DRB1^∗^03:01:01-DQB1^∗^02:01:01. The remainder were heterozygous combinations between this HLA-DR3 haplotype and HLA-DR4; one with HLA-DRB1^∗^04:01:01 (F14-I.i), one with HLA-DRB1^∗^04:02:01 (F22-1.i) and the third with HLA-DRB1^∗^04:05:01 (F24-i.ii). All three HLA-DR4 subtypes were linked to HLA-DQB1^∗^03:02:01:01. The other parent in these 4 families carried only one potential diabetogenic haplotype. The haplotype HLA -DRB1^∗^03:01:01-HLA-DQB1^∗^02:01:01 were the same in all four parents. Of note, none of the 10 parents from the 5 families were diagnosed with diabetes.

## Discussion

This study examined 5 families from the UAE with at least one T1D proband using segregation analysis of HLA alleles. There did not appear to be any recombinant haplotypes. To the best of our knowledge, this is the first time, the classical association between the diabetogenic HLA-DR4 and HLA-DR3 haplotypes has been reported in citizens of the UAE.

There were 3 allelic subtypes for HLA-DR4 identified in these 5 families. The HLA-DRB1^∗^04:01 in family F14 is consistent with those identified in previous association studies in Tunisia and Bahrain (Stayoussef et al., 2009a, b). There were two other subtypes, HLA-DRB1^∗^04:02 and HLA-DRB1^∗^04:05, which have also been linked to the disease in European (Noble, 2015) and Japanese (Kawabata et al., 2002) populations, respectively. The HLA-DRB1^∗^04:05 allele in families F2 and F24 has also been identified as a susceptible allele in populations of the West Asia, namely the non-Askenzi Jews and Arabs (Kwon et al., 2001) and Egyptians (El-Amir et al., 2015). In fact, the rare combination, which is HLA -DRB1^∗^04:05-DQB1^∗^02, that confers risk to T1D was identified in an Egyptian population (El-Amir et al., 2015). The haplotype HLA -DRB1^∗^04:05-DQB1^∗^03:02 in family F24 has also been previously described in T1D patients from Morocco (Drissi Bourhanbour et al., 2015).

There are a number of notable findings in this study. For example, all three offspring in family F14 (Figure 2A) shared both haplotypes for the entire MHC. Specifically, the 3 offspring carried diabetogenic haplotypes previously described in Arabs, namely HLA -DRB1^∗^04:01:01-DQB1^∗^03:02:01 and HLA-DRB1^∗^03:01:01-DQB1^∗^02:01:01.

Secondly, in all five families studied, nine of the 10 parents carried at least one haplotype that has been previously associated with T1D, thus potentially increasing their risk of developing disease. With the exception of family F2, one parent had two T1D risk haplotypes and the second carried one haplotype known to be associated with the disease. Of the 4 parents with 2 disease haplotypes, one was homozygous for HLA -DRB1^∗^03:01:01-DQB1^∗^02:01:01 (F23-I.ii), and 3 carried a heterozygous combination of this HLA-DR3 haplotype and a HLA-DR4 haplotype: one with HLA-DRB1^∗^04:01:01 (F14-I.i), one with HLA-DRB1^∗^04:02:01 (F22-I.ii) and the third with HLA-DRB1^∗^04:05:01 (F24-I.ii). All these HLA-DR4 subtypes were linked to HLA-DQB1^∗^03:02:01:01. The other parent carried only one potential diabetogenic haplotype, HLA -DRB1^∗^04:05:01-DQB1^∗^02:02:01:02 (F2-I.i).

In summary, six out of 11 (55%) individuals with the HLA- DRB1^∗^04-DQA1^∗^03-DQB1^∗^02 haplotype in the five families developed T1D. In comparison, four of 20 (20%) individuals with the HLA- DRB1^∗^03-DQA1^∗^05-DQB1^∗^02 haplotype, and 1 of 2 (50%) with HLA- DRB1^∗^04-DQA1^∗^03-DQB1^∗^03 in the five families developed T1D. This incomplete degree of penetrance for the putative diabetogenic haplotypes is somewhat similar to the overall penetrance of heritable T1D where, on average, only 50% of monozygotic twins of T1D patients, 50% of the offspring of two parents with T1D, and 15-20% of HLA-identical siblings of patients ever get the disease (Salvetti et al., 2000; Redondo et al., 2008; Jerram and Leslie, 2017; Alper et al., 2019). Furthermore, only 5–7 percent of all siblings of patients get the disease, although this may vary depending on populations and ethnicity that have been surveyed (Alper et al., 2019).

Interaction(s) between the candidate disease gene in the MHC class II region and genes in other regions could also explain the apparent incomplete penetrance of the HLA-DR4 and HLA-DR3 haplotypes. Using recombinant mapping and applying this to the highly conserved AH8.1 (HLA -B8-DR3-DQ2), Degli-Esposti et al. (1992a) suggested that both HLA and non-HLA genes are involved in conferring susceptibility, and the region between HLA-B8 and the BAT3 gene may contain some relevant genes. This part of the MHC encodes several genes of immunological relevance including TNFA, the MIC genes and the genes that encode the complement proteins. Coincidently, a portion of AH8.1 was identified in 4 of the 20 haplotypes (20%) that were characterized in this study. Two of these 4 carried the HLA-A^∗^26 allele (Table 2) and has been designated as AH8.2 (Witt et al., 2002), commonly found in Asian Indians. Interestingly, a study by Hajeer et al. (2013) reported that AH8.2 (although with HLA-A^∗^24:02:01:01) was likely one of the most frequent HLA haplotypes in Saudi Arabia.

This AH8.2 has been reported to be associated with autoimmunity and related conditions in Asian Indians (Kaur et al., 2008) including T1D (Mehra et al., 2002; Kanga et al., 2004). This is consistent with admixture analysis which has previously identified Central South Asian genetic lineages in the UAE population (Tay et al., 2020).

Previously, data generated from this laboratory estimated that the inferred haplotype of HLA -A^∗^02-C^∗^06-B^∗^50 was the most frequent 3-locus haplotype in Arabs of the UAE (Kulski et al., 2019). Similarly a study describing the HLA haplotype profile of Kuwaiti Arabs found that HLA -A02:01g-C^∗^06:02-B^∗^50:01-DRB1^∗^07:01-DQB1^∗^02:01g was the most common 5-locus haplotype (Ameen et al., 2020). In this study, there is evidence for this class I component of this haplotype (HLA -A^∗^02-C^∗^06-B^∗^50), extending from class I to class II, namely HLA -C^∗^06:02:01:02-B^∗^50:01:01-DRB1^∗^03:01:01-DQB1^∗^02:01:01. Three independent examples carried this allelic combination (see Table 2). This combination of MHC genes designated AH50.2 (HLA -C^∗^06:02-B^∗^50-DRB1^∗^03:01-DQ2) has been characterized as a T1D associated HLA-DR3 + haplotype that is unique to Asian Indians (Mehra et al., 2007; Kumar et al., 2012). As mentioned, there were no recombinant haplotypes in the 24 meiosis examined in these five families, reflecting the stability of these haplotypes over genomic distances of at least 3.15 megabases (from HLA-A to HLA-DPB1). This highlights the importance of family studies for associating haplotypes to complex disease.

Pedigree-based allele and haplotype phasing are key for understanding the genomic architecture of the population, which is an important step before conducting genetic association studies. Most analysis in case-control studies compare genetic differences between patients and unrelated controls. This strategy generates multiple associated alleles (or markers). However, this method is potentially misleading as it is not known which of those alleles are risk alleles and which are only markers of the differing subpopulations from which the individual (patient/control) originated (Raum et al., 1984; Madsen et al., 2011). In contrast, pedigree-based association studies provide means to minimize subpopulation genetic variation (Alper and Larsen, 2017; Alper et al., 2019). The affected family-based control (AFBAC) method can be used to remove a significant element of ambiguity in hidden population difference which affects the standard case-control method (Alper and Larsen, 2017). Statistical analysis is not presented herein due to the insufficient number of families and will be attempted when more data are available.

The study was conceived with the aim to improve our understanding of the role of genetics of T1D in UAE patients of Middle Eastern descent and stimulate further study in this area. Despite the small sample size, we identified two haplotypes (AH8.2 and AH50.2) that have previously been associated with T1D in a neighboring Indian population.

This presence of these haplotypes in the UAE population is not unexpected since admixture between populations of central south Asia and the Middle East has occurred, giving rise to the contemporary population that is now reside in the UAE (Tay et al., 2020). Future assessment of T1D susceptibility should consider multiple genetic risk factors that are involved along with the awareness that external factors may have a role in the discordance of T1D occurrence as observed in the families presented in this study. Genetic risk score (GRS) models including GRS (Redondo et al., 2018), GRS2 (Sharp et al., 2019), and combined risk score (CRS) (Redondo et al., 2018) have emphasized the fact that environmental factors and ethnicity can potentially mediate different genetic associations which require scores adjustment for the prediction and classification of T1D (Sharp et al., 2019). Further, there are more than 50 coding genes outside the MHC genomic region that have been associated with T1D (Todd, 2010) and should be factored into future analyses. Most of these could be accessed and surveyed as data sets (Burren et al., 2010; Ghoussaini et al., 2021) at ‘‘The Open Targets Genetics Portal’’^1^. Finally, despite only representing 5 families, this study is important as it represents the first description of HLA haplotypes defined by segregation analysis in T1D families from the UAE.

## Data Availability Statement

The data described in this article is deposited in the NCBI BioSample database under the accession numbers SAMN18043501–SAMN18043522.

## Ethics Statement

The studies involving human participants were reviewed and approved by the Mafraq Hospital IRB Ethics Committee. Written informed consent to participate in this study was provided by the participants’ legal guardian/next of kin.

## Author Contributions

HA, AD, and GT conceived the study. AM and ZB developed and carried out the laboratory assays used in the study. AA were responsible for recruitment of the patients contributed data to the study by collecting demographic data. HAN and AA analyzed the data and constructed the figures. HA, GT, and AA provided critical review during manuscript preparation. All authors on the primary list contributed to the data interpretation or critically reviewed the manuscript and approved the final manuscript for submission.

## Conflict of Interest

The authors declare that the research was conducted in the absence of any commercial or financial relationships that could be construed as a potential conflict of interest.

## Footnotes

1 https://genetics.opentargets.org
